# Venous thromboembolism chemoprophylaxis in geriatric trauma patients with isolated severe traumatic brain injury

**DOI:** 10.1007/s00068-023-02299-5

**Published:** 2023-06-12

**Authors:** Freeman Condon, Areg Grigorian, Dylan Russell, Demetrios Demetriades

**Affiliations:** 1 Division of General Surgery, Tripler Army Medical Center, Honolulu, HI USA; 2 Division of Trauma and Surgical Critical Care, University of Southern California, Los Angeles, CA USA; 3 Department of Surgery, University of California, Irvine, 333 City Blvd W, Orange, CA 92868 USA

**Keywords:** Traumatic brain injury, Venous thromboembolism, Geriatrics, Trauma, VTE prophylaxis

## Abstract

**Purpose:**

Low-molecular-weight-heparin (LMWH) has been shown to be associated with a decreased risk of venous thromboembolism (VTE) and mortality compared to unfractionated heparin (UH) in severe traumatic brain injury (TBI). The aim of this study was to see if this association persists among a subset of patients, namely elderly patients with isolated TBI.

**Methods:**

This Trauma Quality Improvement Project (TQIP) database study included patients ≥ 65 years old with severe TBI (Abbreviated injury score [AIS] ≥ 3) that received either LMWH or UH for VTE prophylaxis. Patients with associated severe injuries (extracranial AIS ≥ 3), transferals, deaths < 72-h, hospitalization < 2 days, VTE chemoprophylaxis other than UH or LMWH, or with a history of bleeding diathesis were excluded. The association between VTE, deep vein thrombosis (DVT), and pulmonary embolism (PE) with VTE chemoprophylaxis was analyzed with multivariable analysis, subset analyses of different grades of AIS-head injury, and a 1:1 matched LWMH:UH cohort of patients.

**Results:**

Out of 14,926 patients, 11,036 (73.9%) received LMWH. Multivariate analysis showed that patients receiving LMWH had a decreased risk of mortality (OR 0.81, 95% CI 0.67–0.97, *p* < 0.001) but a similar risk of VTE (OR 0.83, 95% CI 0.63–1.08). Analysis according to head-AIS showed that LMWH was associated with a decreased risk of PE in patients AIS-3 but not in AIS 4 or 5. In a 1:1 matched cohort of LMWH:UH patients, the risk of PE, DVT and VTE were all similar but LMWH continued to be associated with a decreased risk of mortality (OR 0.81, CI 0.67–0.97, *p* = 0.023).

**Conclusion:**

LMWH was associated with a decreased risk of overall mortality and reduced risk of PE compared to UH among geriatric patients with a severe head injury.

## Introduction

With an aging population, nearly one-third of trauma activations will involve a geriatric patient [1, 2]. Relative to younger patients, geriatric patients are more likely to present after a fall, which is the most common etiology leading to traumatic brain injury (TBI) in the geriatric population [3]. TBI is also the most common cause of traumatic death in the United States with over 60,000 deaths annually and is associated with significant morbidity including venous thromboembolism (VTE) which can occur in up to 20% of TBI patients admitted to the intensive care unit [4-6]. The injured brain releases inflammatory products including cytokines and procoagulants leading to a hypercoagulable state [7-9]. Given the higher risk of VTE, preventive efforts are of paramount importance. The safety of VTE chemoprophylaxis in the setting of TBI has been previously demonstrated and the early initiation (< 48 h) is associated with improved outcomes [10, 11].

Low molecular weight heparin (LMWH) has previously been shown to be superior to unfractionated heparin (UH) with respect to mortality and VTE-related complications in the severe traumatic brain-injured patients [12]. This may be due to the neuroprotective properties of LMWH [13, 14].However, the safety profile of VTE chemoprophylaxis and the superiority of LMWH compared to UH in the elderly population has not been fully evaluated. Slower pharmacokinetics as well as a higher incidence of renal disease in the elderly may change the response to VTE chemoprophylaxis in elderly patients with TBI [15]. We, therefore, sought to determine if the superiority of LMWH vs. UH in TBI persisted when examining a cohort of geriatric patients (age ≥ 65 years old) with isolated severe head injuries. We hypothesized that the previously observed benefits of LMWH in severe TBI would persist in the geriatric population.

## Methods

The Trauma Quality Improvement Program (TQIP) database was queried from 2017 to 2019. We identified patients for a retrospective 1:1 propensity score-matched case–control study. Patients ≥ 65 years old with severe TBI who received either LMWH or UH for VTE chemoprophylaxis were identified. Severe TBI was defined as an Abbreviated Injury Scale (AIS) grade of three or higher. Patients with serious injuries outside the head (extracranial AIS ≥ 3) were excluded. Patients with minor or moderate extracranial injuries were not excluded as these injuries are unlikely to contribute to a risk of VTE formation or mortality. Additionally excluded were deaths within 72 h, transfers, hospitalization < 2 days, patients on pre-injury heparin or LMWH, choice of VTE prophylaxis other than UH or LMWH, and history of bleeding diathesis. Due to the robust data capture associated with the TQIP program, patients with incomplete data were very rare. We included this small number of incomplete data sets in our analysis. We then compared patients receiving LMWH to patients receiving UH. The primary outcome was mortality, and the secondary outcome was the development of in-hospital VTE including deep vein thrombosis (DVT) and pulmonary embolus (PE). Due to our use of a de-identified database in this retrospective review, the study was deemed except from institutional review by the Institutional Review Board of the University of Southern California. This study was designed and conducted in adherence to the principles of protection of human subjects set forth in the declaration of Helsinki. Additionally, the study was designed and written with adherence to the STROBE statement for strengthening the results of observational studies in epidemiology.

We collected demographic information including age, sex, hypotension on admission, blood transfusion requirement, need for any surgical intervention including craniectomy/craniotomy within the first 24 h, time to VTE chemoprophylaxis, injury severity score, grade of AIS-head, and comorbid conditions. Frequency statistics were performed for all variables of interest. A chi-square test and Mann–Whitney-*U* test were used to compare categorical and continuous variables, respectively. Categorical data were reported as percentages and continuous data were reported as medians with interquartile range or as means with standard deviation.

We sought to examine the relationship between the choice of VTE chemoprophylaxis and the development of VTE or death. We did this using three distinct analyses including a multivariable logistic regression analysis controlling for known predictors of both VTE and mortality. These variables were carefully chosen after a review of the literature and discussion among coauthors [16, 17]. Covariates with statistical significance (*p* < 0.20) were then included in a hierarchical multivariable logistic regression model and the adjusted risk for mortality was reported with an odds ratio (OR) and 95% confidence intervals (CI). All *p*-values were two-sided, with a statistical significance level of < 0.05. The second analysis included three separate sub-analyses in patients with different grades of isolated head injury including AIS-head grade 3, 4 and 5. And the third analysis involved propensity-score matched analysis. Matched variables included age ≥ 75 years old, sex, GCS, ISS, VTE prophylaxis start date, AIS-head grade, blood product transfusion, major surgery, hypotension on admission, craniectomy/craniotomy, and comorbid conditions. Patients with similar propensity scores were matched in a 1:1 ratio. We included in our analysis only those cases that were within 0.001 of the estimated logit [18]. All analyses were performed with IBM SPSS Statistics for Windows (Version 28, IBM Corp., Armonk, NY).

## Results

Of 14,926 patients, 11,036 (73.9%) received LMWH. Compared to patients receiving UH, those receiving LMWH were less likely to be hypotensive on admission (1.3% vs. 1.9%, p = 0.005) but were more likely to have a grade-5 AIS head injury (25.7% vs. 19.0%, *p* < 0.001—Table 1). Compared to UH, patients receiving LMWH had a similar rate of VTE (2.0% vs. 2.4%, *p* = 0.164) including DVT (1.7% vs. 1.9%, *p* = 0.380). However, patients receiving LMWH had a lower rate of PE (0.4% vs. 0.7%, *p* = 0.016). Most patients in both groups were started on VTE chemoprophylaxis on or after hospital day four (LMWH 47.3%, UH 39.0%, *p* < 0.001). After controlling for known risk factors, the risk of VTE was similar for both groups (OR 0.83, CI 0.63–1.08, *p* = 0.171, Table 2). Initiation of VTE prophylaxis on or after hospital day four was associated with a significantly increased risk of VTE (OR 2.71, CI 1.85–3.97, *p* < 0.001) relative to initiation prior to that point. Other factors that significantly increased the risk of VTE formation were undergoing surgical intervention (OR 2.33, CI 1.80–3.01, *p* < 0.011), male sex (OR 1.55, CI 1.20–2.01 *p* < 0.001), GCS < 9 on presentation (OR 1.84, CI 1.32–2.55, *p* < 0.001), and pre-trauma hypertension (OR 1.39, CI 1.05–1.81, *p* < 0.020) (Table 3). There was no difference in VTE when prophylaxis was started 3 days after the injury as compared with on the first or second day (*p* = 0.378).

**Table 1:** Bivariate analysis of characteristics of geriatric TBI patients receiving LMWH or UH for VTE prophylaxis

Characteristic	LMWH (*n* = 11,036)	UH (*n* = 3890)	*p*-value
Hypotensive on admission	72 (1.9%)	137 (1.3%)	0.005
Male	6232 (56.5%)	2238 (57.5%)	0.246
RBC Transfusion	298 (2.7%)	86 (2.2%)	0.097
Any surgical intervention	2212 (20.0%)	814 (20.9%)	0.239
Age > 75	6222 (56.4%)	2427 (62.4%)	< 0.001
Craniotomy/craniectomy within first 24 h	123 (1.1%)	58 (1.5%)	0.065
Time to prophylaxis (days)			< 0.001
≤ 2	2851 (25.9%)	1096 (28.2%)	
3	2951 (26.8%)	1275 (32.8%)	
≥ 4	5216 (47.3%)	1517 (30%)	
Injury severity score			< 0.001
≤ 9	883 (8.0%)	378 (9.7%)	
10–15	4020 (36.4%)	1214 (31.2%)	
16–24	3998 (36.2%)	1290 (33.2%)	
≥ 25	2134 (19.3%)	1008 (25.9%)	
Total GCS			0.455
14–15	8173 (77.4%)	2848 (78.3%)	
9–13	1449 (13.7%)	470 (12.9%)	
3–8	938 (8.9%)	321 (8.8%)	
AIS-Head (grade)			< 0.001
3	5719 (51.8%)	1728 (44.4%)	
4	3221 (29.2%)	1163 (29.9%)	
5	2091 (19%)	998 (25.7%)	
Comorbidities			
Congestive heart failure	829 (7.6%)	427 (11.3%)	< 0.001
Cirrhosis	139 (1.3%)	58 (1.5%)	0.239
COPD	989 (9%)	403 (10.6)	0.004
Stroke	849 (7.8%)	328 (8.6%)	0.087
Diabetes	2943 (26.9%)	1223 (32.0%)	< 0.001
Hypertension	7165 (65.2%)	2760 (71.8%)	< 0.001
Chronic kidney disease	189 (1.7%)	228 (6.0%)	< 0.001
Myocardial infarction	184 (1.7%)	74 (2.0%)	0.272

*LMWH* low molecular weight heparin, *UH* unfractionated heparin, *RBC* red blood cell,* GCS* Glasgow coma scale,* AIS* Abbreviated Injury Scale, *COPD* Chronic Obstructive Pulmonary Disease

**Table 2:** Multivariable logistic regression analysis for risk of venous thromboembolism in geriatric trauma patients with isolated severe traumatic brain injury

Risk factor	OR	CI	*p*-value
LMWH vs. UH	0.83	0.63–1.08	0.171
Hypotensive on admsision	1.93	1.00–3.76	0.053
Blood Transfusion	1.57	0.96–2.60	0.075
Any surgical intervention	2.33	1.80–3.01	< 0.001
Male Sex	1.55	1.20–2.01	< 0.001
Craniotomy/craniectomy within first 24 h	1.09	0.54–2.22	0.809
Age > 75 years	1.16	0.90–1.47	0.248
Time to Prohylaxis (days)			
≤ 2	REFERENCE		
3	1.22	0.78–1.91	0.378
≥ 4	2.71	1.85–3.97	< 0.001
Injury Severity Score			
< 9	REFERENCE		
9–15	0.853	0.49–1.47	0.568
16–24	1.30	0.68–2.51	0.43
≥ 25	2.85	0.71–11.40	0.138
Total GCS			
14–15	REFERENCE		
9–13	1.48	1.08–2.01	0.13
3–8	1.84	1.32–2.55	< 0.001
AIS-Head (grade)			
3	REFERENCE		
4	0.82	0.50–1.34	0.430
5	0.52	0.14–1.91	0.321
CHF	1.01	0.66–1.55	0.968
COPD	1.05	0.71–1.58	0.782
Diabetes	1.11	0.85–1.44	0.454
Hypertension	1.38	1.05–1.81	0.020
CKD	0.956	0.48–1.90	0.899

*LMWH* low molecular weight heparin, *UH* unfractionated heparin, *GCS* Glasgow Coma Scale, *AIS* Abbreviated Injury Scale, *CHF* Congestive Heart Failure, *COPD* Chronic Obstructive Pulmonary Disease, *CKD* Chronic Kidney Disease

**Table 3:** Multivariable logistic regression analysis for risk of mortality in geriatric trauma patients with isolated severe traumatic brain injury

Risk Factor	OR	CI	*p*-value
LMWH vs. UH	0.77	0.66–0.91	0.001
Hypotensive on admsision	1.87	1.21–2.9	0.005
Blood Transfusion	1.67	1.23–2.29	0.001
Any surgical intervention	1.60	1.36–1.88	< 0.001
Male Sex	1.40	1.20–1.63	< 0.001
Craniotomy/craniectomy within first 24 h	1.98	1.35–2.884	< 0.001
Age > 75 years	1.86	1.60–2.18	< 0.001
Time to Prohylaxis (days)			
≤ 2	REFERENCE		
3	1.03	.882–1.29	0.793
≥ 4	1.24	1.20–1.77	0.036
Injury Severity Score			
< 9	REFERENCE		
9–15	0.86	0.60–1.28	0.494
16–24	1.09	0.67–1.77	0.729
≥ 25	1.94	0.772–5.25	0.188
Total GCS			
14–15	REFERENCE		
9–13	2.51	2.13–3.08	< 0.001
3–8	4.67	3.86–5.67	< 0.001
AIS-Head (grade)			
3	REFERENCE		
4	1.41	0.98–2.04	0.066
5	1.75	0.69–4.48	0.237
CHF	1.52	1.20–1.91	< 0.001
COPD	1.21	0.95–1.53	0.124
Diabetes	1.18	1.00–1.38	0.048
Hypertension	0.83	0.70–0.97	0.018
CKD	1.81	1.27–2.57	< 0.001

*LMWH* low molecular weight heparin, *UH* unfractionated heparin, *GCS* Glasgow Coma Scale, *AIS* Abbreviated Injury Scale, *CHF* Congestive Heart Failure, *COPD* Chronic Obstructive Pulmonary Disease, *CKD* Chronic Kidney Disease

Patients receiving LMWH had a lower risk of death (OR 0.77, CI 0.66–0.91, *p* = 0.001, Table 3) compared to the patients receiving UH. Other factors associated with increased risk of mortality were hypotension on admission, need for transfusion or surgery, craniotomy or craniectomy within 24 h, age > 75. Pre-trauma CHF, diabetes, hypertension, and CKD were all associated with increased risk of mortality but COPD was not (Table 3). Additionally GCS < 14 on arrival and AIS of 5 were independent predictors of mortality (Table 3).

Subgroup analysis by AIS grade demonstrated no difference in VTE incidence regardless of LMWH vs UH. It did, however, demonstrate a decreased risk of death in AIS grade 4 (OR 0.72, CI 0.53–0.97, *p* = 0.028) and 5 (OR 0.74, CI 0.59–0.92, *p* = 0.006), but not in AIS 3 (*p* > 0.05) (see Table 4).

**Table 4:** Multivariable logistic regression analysis for risk of venous thromboembolism and mortality in geriatric trauma patients with isolated traumatic brain injury (LMWH vs. UH) separated by AIS-head-grade

Risk factor	OR	CI	*p*-value
AIS-Head Grade 3			
VTE	0.83	0.52–1.32	0.423
Mortality	0.82	0.59–1.13	0.224
AIS-Head Grade 4			
VTE	0.64	0.40–1.02	0.059
Mortality	0.72	0.53–0.97	0.028
AIS-Head Grade 5			
VTE	0.94	0.62–1.43	0.761
Mortality	0.74	0.59–0.92	0.006

Controlled for hypotension on admission, blood transfusion, any surgical intervention, male sex, craniectomy/craniotomy within first 24-h, age ≥ 75-years-old, time to prophylaxis, injury severity score, Glasgow coma scale, and AIS-head grade

*LMWH* low-molecular-weight-heparin, *UH* unfractionated heparin, *AIS* abbreviated injury scale, *VTE* venous thromboembolism

We also performed a propensity-matched cohort analysis. There was no difference in matched variables (all *p* > 0.05). The risk of VTE was similar in both groups (*p* = 0.209) but patients receiving LMWH had a decreased risk of death (OR 0.81 CI 0.067–0.97, *p* = 0.023) compared to patients receiving UH (see Table 5).

**Table 5:** Logistic regression analysis for risk of venous thromboembolism and mortality in geriatric trauma patients with isolated traumatic brain injury in a 1:1 matched cohort of LMWH:UH patients

Risk factor	OR	CI	*p*-value
VTE	0.81	0.58–1.13	0.209
Mortality	0.81	0.67–0.97	0.023

*LMWH* low-molecular-weight-heparin, *UH* unfractionated heparin, *AIS* abbreviated injury scale, *VTE* venous thromboembolism

## Discussion

Our findings demonstrate that the previously described association between LMWH and decreased mortality relative to UH does not persist in a geriatric trauma population presenting with isolated severe head trauma. The risk of VTE is similar regardless of receiving LMWH or UH and this association was studied using three distinct analyses. We did, however, find that LMWH is associated with a reduced risk of death in patients with severe and critical TBI.

A previous study by Benjamin, et. al., demonstrated the superiority of LMWH to UH with respect to DVT and PE formation [12]. Despite these findings, significant variability in VTE chemoprophylaxis among trauma centers persists [19]. Some reasons for hesitation in the adoption of LMWH are the result of the shorter half-life of UH and the presence of a complete reversal agent in protamine sulfate [20, 21]. The prevention of VTE in trauma patients must be balanced against the risk of precipitating bleeding complications. Among severe TBI patients, this risk is of particular importance, as even small progressions of intracranial bleeding can result in catastrophic sequalae. The use of LMWH has received historical criticism arising from a concern for the progression of intracranial hemorrhage [22]. Despite these concerns, subsequent studies demonstrate the safety of early initiation of LMWH in severe TBI [10, 23]. A potential additional concern with respect to the use of LMWH in the geriatric patient arises from its need for renal clearance, and a concern for precipitating acute kidney injury. Despite this concern, the use of LMWH has been safely demonstrated even in geriatric patients with creatinine clearance < 30 mL/min when dosed with the use of Anti-Xa levels [24]. Additionally, there is increasing momentum towards the preferential use of LMWH over UH in geriatric non-trauma patients [25].

This study demonstrates that the mortality benefit associated with LMWH persists when looking at the subset of trauma patients in the geriatric cohort. Interestingly, this mortality benefit persists despite similar rates of DVT, PE, and VTE between UH and LMWH groups. This suggests that additional factors must account for the decreased risk of death. One mechanism for this protective effect proposed in the literature is a neuroprotective effect of LMWH mediated by decreased cerebral edema in TBI [13]. In fact, a recent randomized clinical trial demonstrated the effectiveness of LMWH as a treatment for TBI [26]. If this effect is borne out in future studies, it would provide an additional robust incentive for the use of LMWH in the TBI patient.

An additional interesting finding of this study is the significant effect of the timing of prophylaxis on VTE incidence. There was no observable difference in VTE incidence whether prophylaxis was started on the first, second, or third day. If prophylaxis was not initiated until day four, however, there was a significantly increased risk of VTE (OR 2.77, CI 1.89–4.05, *p* < 0.001). Though a similar pattern is evident with respect to mortality, this trend did not achieve significance. These findings confirm what we already know- that when it comes to initiating post-injury VTE prophylaxis, hours matter.

Our study is chiefly limited by its retrospective and observational nature. While the numbers of missing data in the set was low, the database does not capture 100% of reportable outcomes. As a database study, these findings are limited by the tendency towards underreporting of outcomes [27]. While instances of VTE are likely somewhat underreported due to loss of follow-up of trauma patients after discharge, there is no reason to suspect that this would bias towards any one chemotherapeutic agent. Though we did control for differences using cohort matching, Table 1 illustrates that there were some fundamental differences in the groups of patients who received UH vs LMWH (eg. slightly more hypotension on admission in the LMWH group)Additionally there are no specific data regarding the progression of intracranial hemorrhages. Nonetheless, the study is robustly powered given the numbers of patients captured by the TQIP database. Given the heterogeneity of TQIP enrollees and the extremely broad pool from which data is collection, one of the studies key strengths is strong external validity. Additionally, the persistence of a mortality benefit in the 1:1 matched LMWH:UH heparin cohort suggests that the observed outcomes are truly due to association with the choice of VTE prophylaxis agent.

## Conclusion

The geriatric patient is not simply an “older adult.” There are fundamental changes in physiology, pharmacokinetics, pathogenesis and progression of the disease, for which one must account. Nonetheless, concerns regarding the use of LMWH in the elderly trauma patient do not appear to have a causal basis in the data. This study demonstrates that among geriatric TBI patients early use of LMWH over UH is associated with decreased mortality overall and a similar risk of VTE. Future research could confirm these findings using a prospective model which would address some of the limitations of this study. Additionally, future studies could aim to address whether these findings persist in the multiply injured geriatric patient with both TBI and extracranial injury.

## References

[1] Kozar RA, Arbabi S, Stein DM, Shackford SR, Barraco RD, Biffl WL, Brasel KJ, Cooper Z, Fakhry SM, Livingston D, Moore F, Luchette F (2015). Injury in the aged: Geriatric trauma care at the crossroads. J Trauma Acute Care Surg.

[2] Amoako J, Evans S, Brown NV, Khaliqdina S, Caterino JM (2019). Identifying predictors of undertriage in injured older adults after implementation of statewide geriatric trauma triage criteria. Acad Emerg Med.

[3] Thompson HJ, McCormick WC, Kagan SH (2006). Traumatic brain injury in older adults: epidemiology, outcomes, and future implications. J Am Geriatr Soc.

[4] Skrifvars MB, Bailey M, Presneill J, French C, Nichol A, Little L, Duranteau J, Huet O, Haddad S, Arabi Y, McArthur C, Cooper DJ, Bellomo R, EPO-TBI investigators and the ANZICS Clinical Trials Group (2017). Venous thromboembolic events in critically ill traumatic brain injury patients. Intensive Care Med.

[5] Prabhakaran K, Gogna S, Lombardo G, Latifi R (2020). Venous thromboembolism in geriatric trauma patients-risk factors and associated outcomes. J Surg Res.

[6] Centers for Disease Control and Prevention. National Center for Health Statistics: Mortality data on CDC WONDER. https://wonder.cdc.gov/mcd.html Accessed 15 Aug 2022

[7] Van Gent JM, Bandle J, Calvo RY, Zander AL, Olson EJ, Shackford SR, Peck KA, Sise CB, Sise MJ (2014). Isolated traumatic brain injury and venous thromboembolism. J Trauma Acute Care Surg.

[8] Drake TA, Morrissey JH, Edgington TS (1989). Selective cellular expression of tissue factor in human tissues. Implications for disorders of hemostasis and thrombosis. Am J Pathol.

[9] Fisher MJ (2013). Brain regulation of thrombosis and hemostasis: from theory to practice. Stroke.

[10] Mesa Galan LA, Egea-Guerrero JJ, Quintana Diaz M, Vilches-Arenas A (2016). The effectiveness and safety of pharmacological prophylaxis against venous thromboembolism in patients with moderate to severe traumatic brain injury: a systematic review and meta-analysis. J Trauma Acute Care Surg.

[11] Koehler DM, Shipman J, Davidson MA, Guillamondegui O (2011). Is early venous thromboembolism prophylaxis safe in trauma patients with intracranial hemorrhage. J Trauma.

[12] Benjamin E, Recinos G, Aiolfi A, Inaba K, Demetriades D (2017). Pharmacological thromboembolic prophylaxis in traumatic brain injuries: low molecular weight heparin is superior to unfractionated heparin. Ann Surg.

[13] Li S, Eisenstadt R, Kumasaka K, Johnson VE, Marks J, Nagata K, Browne KD, Smith DH, Pascual JL (2016). Does enoxaparin interfere with HMGB1 signaling after TBI? A potential mechanism for reduced cerebral edema and neurologic recovery. J Trauma Acute Care Surg.

[14] Li S, Marks JA, Eisenstadt R, Kumasaka K, Samadi D, Johnson VE, Holena DN, Allen SR, Browne KD, Smith DH, Pascual JL (2015). Enoxaparin ameliorates post-traumatic brain injury edema and neurologic recovery, reducing cerebral leukocyte endothelial interactions and vessel permeability in vivo. J Trauma Acute Care Surg.

[15] Clark NP (2008). Low-molecular-weight heparin use in the obese, elderly, and in renal insufficiency. Thromb Res.

[16] Fu WW, Fu TS, Jing R, McFaull SR, Cusimano MD (2017). Predictors of falls and mortality among elderly adults with traumatic brain injury: A nationwide, population-based study. PLoS ONE.

[17] Javali RH, Krishnamoorthy M, Patil A, Srinivasarangan M, Suraj S (2019). Comparison of injury severity score, new injury severity score, revised trauma score and trauma and injury severity score for mortality prediction in elderly trauma patients. Indian J Crit Care Med..

[18] Austin PC (2011). Optimal caliper widths for propensity-score matching when estimating differences in means and differences in proportions in observational studies. Pharm Stat.

[19] Regner JL, Shaver CN, SWSC Multicenter Trials Group (2019). Determining the impact of culture on venous thromboembolism prevention in trauma patients: a Southwestern Surgical Congress Multicenter trial. Am J Surg.

[20] Paffrath T, Wafaisade A, Lefering R, Simanski C, Bouillon B, Spanholtz T, Wutzler S, Maegele M, Trauma Registry of DGU (2010). Venous thromboembolism after severe trauma: incidence, risk factors and outcome. Injury.

[21] Selby R, Geerts W, Ofosu FA, Craven S, Dewar L, Phillips A, Szalai JP (2009). Hypercoagulability after trauma: hemostatic changes and relationship to venous thromboembolism. Thromb Res.

[22] Kwiatt ME, Patel MS, Ross SE, Lachant MT, MacNew HG, Ochsner MG, Norwood SH, Speier L, Kozar R, Gerber JA, Rowell S, Krishnakumar S, Livingston DH, Manis G, Haan JM (2012). Is low-molecular-weight heparin safe for venous thromboembolism prophylaxis in patients with traumatic brain injury? A Western Trauma Association multicenter study. J Trauma Acute Care Surg.

[23] Jamjoom AA, Jamjoom AB (2013). Safety and efficacy of early pharmacological thromboprophylaxis in traumatic brain injury: systematic review and meta-analysis. J Neurotrauma.

[24] Pellizzari L, Facchinetti R, Corrà L, Sepe A, Fantin F, Fontana G, Zamboni M, Di Francesco V (2018). Can we reliably predict the level of anticoagulation after enoxaparin injection in elderly patients with renal failure?. Aging Clin Exp Res.

[25] Dorobantu M, Bogdan S (2016). Unfractionated heparin or low-molecular-weight heparin in the elderly. Int J Cardiol.

[26] Baharvahdat H, Ganjeifar B, Etemadrezaie H, Farajirad M, Zabihyan S, Mowla A (2019). Enoxaparin in the treatment of severe traumatic brain injury: a randomized clinical trial. Surg Neurol Int.

[27] Hunt JP, Cherr GS, Hunter C, Wright MJ, Wang YZ, Steeb G, Buechter KJ, Meyer AA, Baker CC (2000). Accuracy of administrative data in trauma: splenic injuries as an example. J Trauma.

